# Reviewer acknowledgement 2014

**DOI:** 10.1186/s13601-015-0048-9

**Published:** 2015-02-17

**Authors:** Clive Grattan

**Affiliations:** Norfolk and Norwich University Hospital, Norwich, UK

## Abstract

The editors of *Clinical and Translational Allergy* would like to thank all of our reviewers who have contributed to the journal in Volume 4 (2014).

**Claudia Alessandri**

Italy

**Darío Antolín-Amérigo**

Spain

**Leonardo Antonicelli**

Italy

**Andrew Bartholomaeus**

Australia

**Sevim Bavbek**

Turkey

**Cecilia Berin**

United States of America

**Tilo Biedermann**

Germany

**M.Beatrice Bilò**

Italy

**Carsten Bindslev-Jensen**

Denmark

**Gulbin Bingol Karakoc**

Turkey

**Barbara Bohle**

Austria

**Apostolos Bossios**

Sweden

**Fulvio Braido**

Italy

**Knut Brockow**

Germany

**Indre Butiene**

Lithuania

**Carlo Caffarelli**

Italy

**Virginia Calder**

United Kingdom

**Paloma Campo**

Spain

**Giorgio Walter Canonica**

Italy

**Ozlem Cavkaytar**

Turkey

**Yoon-Seok Chang**

South Korea

**Araceli Díaz Perales**

Spain

**Ibon Eguiluz Gracia**

Norway

**Philippe Eigenmann**

Switzerland

**Filippo Fassio**

Italy

**Penny Fitzharris**

New Zealand

**Bertine Flokstra - de Blok**

Netherlands

**Adam Fox**

United Kingdom

**Venugopal Gangur**

United States of America

**Vanessa Garcia Larsen**

United Kingdom

**Roy Gerth van Wijk**

Netherlands

**Kate Grimshaw**

United Kingdom

**Marion Groetch**

United States of America

**Christoph Grüber**

Germany

**Peter Hellings**

Belgium

**Michihiro Hide**

Japan

**Karin Hoffmann-Sommergruber**

Austria

**Valerie Hox**

Belgium

**Marek Jutel**

Poland

**Leon Knippels**

Netherlands

**Edward Knol**

Netherlands

**George Konstantinou**

United States of America

**Karoline Krause**

Germany

**Annette Kuehn**

Luxembourg

**Moises Labrador-Horrillo**

Spain

**Gideon Lack**

United Kingdom

**Lin-Feng Li**

China

**Dan Lipsker**

France

**Markus Magerl**

Germany

**Nadine Marrouche**

United Kingdom

**Andrea Mikkelsen**

Sweden

**Clare Mills**

United Kingdom

**Pedro Moreira**

Portugal

**Carmen Moreno Aguilar**

Spain

**Hideaki Morita**

Switzerland

**Antonella Muraro**

Italy

**Oscar Palomares**

Spain

**María Pedrosa**

Spain

**Daniel Pérez-Formigo**

Spain

**Constantinos Pitsios**

Greece

**Thomas Platts-Mills**

United States of America

**Laura Polloni**

Italy

**Anna Pomes**

United States of America

**Graham Roberts**

United Kingdom

**Carmen Rondon**

Spain

**Alexandra Santos**

United Kingdom

**Stephan Scheurer**

Germany

**Peter Schmid-Grendelmeier**

Switzerland

**Gianenrico Senna**

Italy

**Milena Sokolowska**

United States of America

**Jelena Stevanovic**

Netherlands

**Luis Taborda-Barata**

Portugal

**Massimo Triggiani**

Italy

**Paul Turner**

United Kingdom

**Eva-Maria Varga**

Austria

**Carina Venter**

United Kingdom

**Carmen Vidal**

Spain

**Margitta Worm**

Germany

**Paraskevi Xepapadaki**

Greece

**S. Tolga Yavuz**

Turkey

**Josefina Zakzuk**

Colombia

